# Uric Acid and Diabetic Retinopathy: A Systematic Review and Meta-Analysis

**DOI:** 10.3389/fpubh.2022.906760

**Published:** 2022-05-31

**Authors:** Yicong Guo, Siyue Liu, Huilan Xu

**Affiliations:** Department of Social Medicine and Health Management, Xiangya School of Public Health, Central South University, Changsha, China

**Keywords:** uric acid, diabetic retinopathy, systematic review, meta-analysis, non-proliferative diabetic retinopathy, proliferative diabetic retinopathy

## Abstract

**Background:**

The relationship between uric acid (UA) and diabetic retinopathy (DR) remains ambiguous, and the results of current studies on the UA levels in patients with DR are conflicting. A meta-analysis was performed to provide a better understanding of the relationship between UA levels and DR.

**Methods:**

PubMed, Web of Science, Embase, and the Cochrane Library databases were searched until December 11, 2021 to identify eligible studies, that compared the UA levels of the case group (patients with DR) and control group (controls with diabetes and healthy participants). The weighted mean difference (WMD) with a 95% confidence interval (CI) was used to evaluate the difference in UA levels between the case and control groups.

**Results:**

Twenty-one studies involving 4,340 patients with DR and 8,595 controls (8,029 controls with diabetes and 566 healthy participants) were included in this meta-analysis. We found that patients with DR had significantly higher UA levels than those in the controls with diabetes (WMD = 36.28; 95% CI: 15.68, 56.89; *P* < 0.001) and healthy participants (WMD = 70.80; 95% CI: 19.85, 121.75; *P* = 0.006). There was an obvious heterogeneity among the 21 studies (*I*^2^ = 97%, *P* < 0.001). Subgroup analyses of different phases of DR showed that UA levels were significantly increased in participants with proliferative diabetic retinopathy (PDR) (WMD = 46.57; 95% CI: 28.51, 64.63; *P* < 0.001) than in controls with diabetes; however, the difference is not statistically significant when comparing UA levels in patients with non-proliferative diabetic retinopathy (NPDR) and controls with diabetes (WMD = 22.50; 95% CI: −6.07, 51.08; *P* = 0.120). In addition, UA levels were higher in participants with a body mass index (BMI) ≥25.0 kg/m^2^ and over 15 years of diabetes. Univariate meta-regression analysis revealed that BMI (*P* = 0.007, Adj *R*^2^ = 40.12%) and fasting blood glucose (FBG) (*P* = 0.040, Adj *R*^2^ = 29.72%) contributed to between-study heterogeneity.

**Conclusions:**

In conclusion, our study provides evidence that UA levels are higher in patients with DR than those in the controls, but this difference is not statistically significant in the early phases. UA might be a potential biomarker for identifying disease severity in patients with DR, rather than predicting the onset of DR among patients with diabetes. However, more prospective and high-quality clinical evidence is required to confirm these present findings.

**Systematic Review Registration:**

https://www.crd.york.ac.uk/PROSPERO/display_record.php?RecordID=297708.

## Introduction

According to the International Diabetes Federation (IDF) estimates of the global prevalence of diabetes mellitus (DM), 700 million (10.9%) people will have diabetes by 2045, representing a 51% increase compared with that in 2019 (1). With the increasing number of people with diabetes, it is foreseeable that the prevalence of diabetic retinopathy (DR) is also expected to increase. DR is a common microvascular complication of diabetes affecting more than 30% of patients with diabetes worldwide and is one of the leading causes of acquired blindness globally in the working-age adult population (2–4). DR is divided into two progressive phases, non-proliferative (earlier) and proliferative (late), and eventually deteriorates into vision-threatening DR (VTDR) (5). The pathogenesis of DR is known as a complex interplay between neuroglial and vascular damage that results from hyperglycemia-induced metabolic oxidative stress, and improving microcirculation of the retina was proven to be effective in preventing the early development of DR (6–9). In addition, previous studies have found that DR may be associated with inflammation and dysregulation of various inflammatory mediators (10–12).

Uric acid (UA) is the final product of purine metabolism and is typically considered the predominant predictor of gout. A UA concentration of 6 mg/dL is recommended as the threshold for the definition of hyperuricemia and as the minimum uricemia target for UA-lowering therapy in patients with gout (13). In addition to being closely linked to gout, increased UA levels have been shown to be associated with the risk of diabetes and some of its complications, such as diabetic peripheral neuropathy and diabetic nephropathy (14–16). Similarly, UA is likely to contribute to DR occurrence. For example, UA has been demonstrated to promote an inflammatory response to release inflammatory factors such as tumor necrosis factor-α (TNF-α), interleukin-6 (IL-6), and C-reactive protein (CRP) (17), and a recent meta-analysis showed that IL-6 was associated with the incidence of DR (18). However, the relationship between UA levels and DR remains ambiguous, and the results of current studies on UA levels in patients with DR are conflicting. Some studies have reported increased UA levels in patients with DR compared with patients with diabetes without DR (19–21), but the results of other studies were different or even opposite (22–25).

No meta-analytical data provided the overall information on this issue. Thus, to obtain a more precise assessment of the association between DR and serum and plasma UA levels and explore the possibility of UA as a predictor for DR in patients with diabetes, we conducted a systematic review and meta-analysis to summarize the current evidence.

## Methods

This systematic review and meta-analysis followed the Preferred Reporting Items for Systematic reviews and Meta-Analyses (PRISMA) guidelines (26). The PRISMA checklist for reporting the meta-analysis results is shown in Supplementary Table 1. The study protocol was registered in the PROSPERO International Prospective Register of Systematic Reviews (CRD42022297708).

### Literature Search

We performed a comprehensive search of PubMed, Web of Science, Embase, and the Cochrane Library databases up to December 11, 2021, to acquire original articles. A combination of keywords and mesh terms was used as a search strategy: (“uric acid” OR “urate” OR “hyperuricemia” OR “serum uric acid”) AND (“diabetic retinopathy” OR “diabetic complication” OR “microvascular complication” OR “DR”). The terms were appropriately adjusted for each database. We also screened the references of relevant studies and reviewed articles to identify additional published and unpublished records.

### Inclusion and Exclusion Criteria

Our meta-analysis included all studies meeting the following explicit criteria: (1) studies were designed as a comparative study, completely involving a case group (patients with diabetes with DR) and control group (patients with diabetes without DR or participants without diabetes); (2) the concentrations of UA (mean and standard deviation) and the number of individuals in each group were available; (3) studies in which UA levels were measured in blood specimens (plasma, serum, or whole blood); and (4) studies were published or written in English.

The exclusion criteria were as follows: (1) case reports, abstracts, and reviews (including systematic reviews and meta-analyses); (2) study protocols, letters, comments, and conference abstracts; (3) experimental or animal studies; and (4) duplicate studies retrieved from various databases.

### Data Extraction and Quality Assessment

For each eligible study, two authors (GY-C and LS-Y) independently extracted the following data: (1) first author's last name, publication year, region of study, the grouping of each study, and sample size; (2) demographic characteristics of participants, including ages, percentage of male participants, body mass index (BMI), types of diabetes, and duration of diabetes; (3) laboratory test results in participants with diabetes such as fasting blood glucose (FBG), glycated hemoglobin A_1c_ (HbA_1c_), total cholesterol (TC), and low-density lipoprotein (LDL); (4) concentrations of UA (mean and standard deviation), and all of the units were converted into μmol/L (1 mg/dL = 59.48 μmol/L); and (5) detecting methods and source of specimen for UA.

The quality of the included studies was assessed using the Newcastle-Ottawa Scale (NOS) for non-randomized studies. NOS is a rating scale in which points are awarded to studies based on selection, comparability, and exposure or outcome, where each study score ranges from 0 to 9 points (27). A study with a total quality score of more than 7 points was considered a high-quality study. Two researchers (GY-C and LS-Y) independently rated the study quality, and differences in ratings between reviewers were resolved by discussion.

### Statistical Analysis

The fixed-effects (or random-effects) inverse-variance model (for continuous data) with the DerSimonian-Laird estimate of tau^2^ was used to pool mean differences (MDs) from all included studies, and the weighted mean difference (WMD) with a 95% confidence interval (CI) was used to evaluate the difference in UA levels between the case and control groups. We generated a forest plot of the differences in UA levels between patients with DR and controls (controls with diabetes and healthy participants were separately compared). Heterogeneity was evaluated using Cochran's Q-statistic test and I-squared (*I*^2^). A value of *I*^2^ of 0–25% represents insignificant heterogeneity, >25% but <50% represents low heterogeneity, >50% but <75% represents moderate heterogeneity, and >75% represents high heterogeneity (46, 47). The *P-*value of the *Q*-test <0.10 was considered statistically significant. If *I*^2^ ≥ 50% and *P* < 0.10, the random-effects model was used; otherwise, the fixed-effects model was applied (48). Subgroup analysis grouped by DR phases [non-proliferative diabetic retinopathy (NPDR) vs. controls with diabetes; proliferative diabetic retinopathy (PDR) vs. controls with diabetes; and PDR vs. NPDR], region (Asia and others), diabetes type (type 1, type 2, and both), duration of diabetes ( ≤ 15 and >15 years), BMI ( ≤ 25 and >25 kg/m^2^), FBG ( ≤ 150 and >150 mg/dL), HbA1c ( ≤ 8.0% and >8.0%), LDL ( ≤ 120 and >120 mg/dL), specimen types (plasma and serum), and quality score (<7 and ≥7) was performed to investigate the differences in studies or participants with different characteristics and explore the origin of heterogeneity. The UA concentrations in patients with DR were stratified (quartiles 1–4: 285.4–307.1; 307.1–333; 333–378.15; and 378.15–505.6 μmol/L) to explore a linear dose-response correlation of the pooled results in patients with DR with different ranges of UA levels. When heterogeneity was high, a univariate meta-regression analysis was performed to identify potential confounding factors and explore the sources of heterogeneity. A sensitivity analysis was performed to evaluate the effect of a particular study on the overall results by omitting one study and combining the remainders in each turn. Egger's (49) and Begg's tests (50) were used to assess potential publication bias, and a visualized funnel plot was performed as a complement.

All statistical analyses were performed using RevMan 5.4 software (The Cochrane Collaboration, Copenhagen, Denmark) and Stata v16.0 (Stata Corp LP, College Station, TX, USA). A two-sided *P* < 0.05 was considered statistically significant except for the Cochran *Q*-test. In our study, all analyses were based on previously published research; therefore, no ethical approval or patient consent was required.

## Results

### Search Results and Study Characteristics

**Figure 1:**
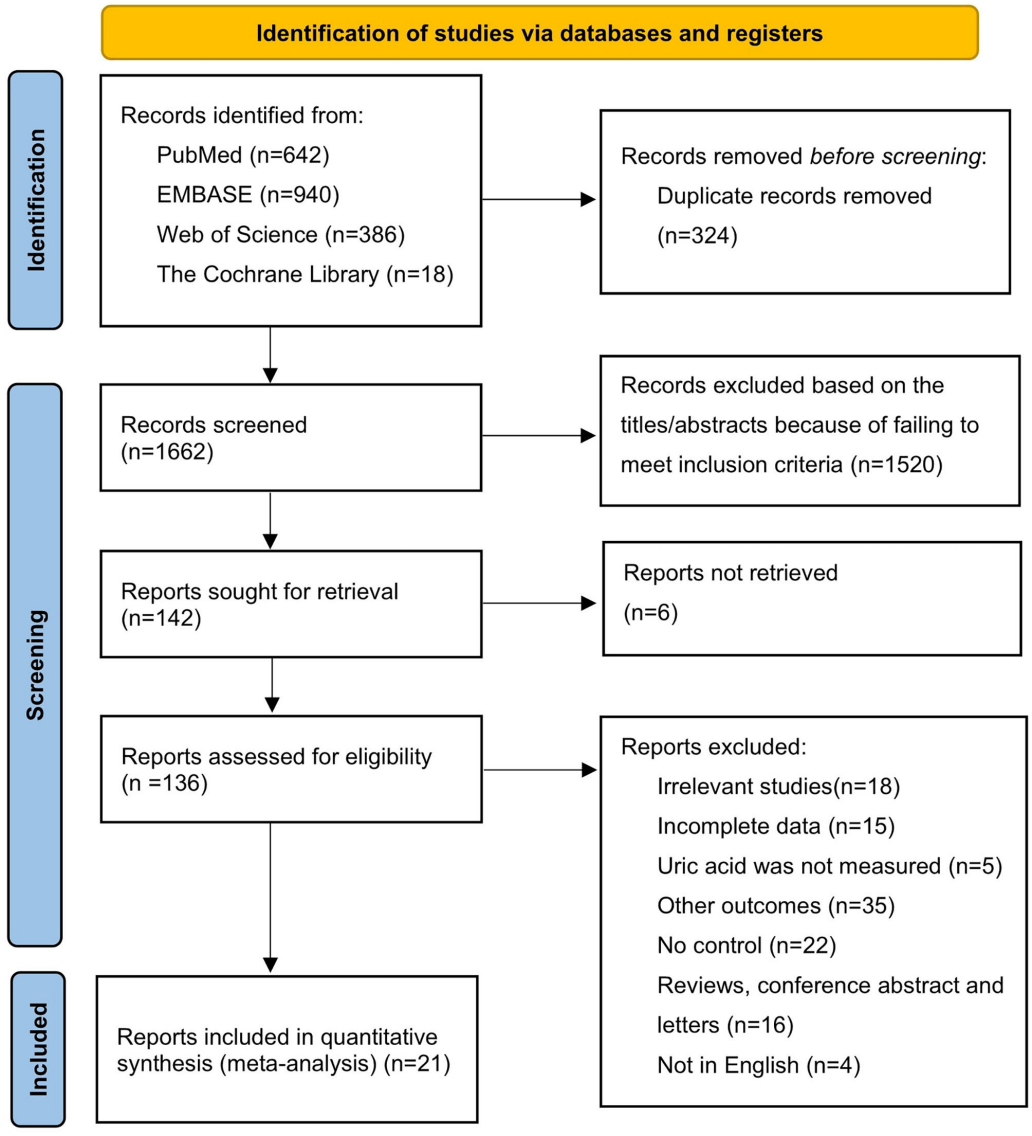
Flow chart of the study selection process.

Our search yielded 1,986 potentially relevant articles in electronic databases: 642 from PubMed, 940 from Embase, 386 from Web of Science, and 18 from the Cochrane Library. After excluding duplicate studies, 1,662 articles were retained. Of the 1,662 studies initially identified, 1,520 were excluded because they failed to meet the inclusion criteria based on title and abstract review. The full texts of the remaining 142 articles were reviewed for eligibility, and 121 articles were excluded for various reasons, such as not being retrieved, irrelevant studies, incomplete data, other outcomes, and a review article or conference abstract. We finally selected a total of 21 qualified articles (20, 22, 23, 28–45) involving 4,340 patients with DR and 8,595 controls (containing 8,029 controls with diabetes and 566 healthy participants) in this meta-analysis. A flowchart of the literature search process is shown in Figure 1.

Table 1 presents the characteristics of the 21 eligible studies, including the first author's last name, publication year, region, diabetes type, specimen type, detection method for UA, grouping of each study, sample size of each group, and the NOS score. Accordingly, 16 studies were conducted in Asia (22, 23, 29–32, 34–38, 40, 42–45), and other studies were conducted in Africa (28, 33, 41), Europe (39), and South America (20). In addition, two studies were based on type 1 diabetes (20, 39) with 2,311 included participants, and the others were based on type 2 diabetes (22, 29–38, 40, 43–45) and both types of diabetes (23, 28, 32, 41, 42). The colorimetric method has been used in most studies to measure UA concentrations, and only one study applied the high-performance liquid chromatography (HPLC) method (34). Except for three studies (28, 34, 39) that measured UA concentrations using plasma, serum was utilized for the measurement of UA concentrations in other studies. Eleven studies (20, 22, 29, 34, 35, 37–40, 43, 44) that scored 7 or higher were considered high quality, and others (23, 28, 30–33, 36, 41, 42, 45) scored from 4 to 6, indicating that the overall quality of the studies was acceptable. The participants' characteristics, including age, sex, and BMI, are summarized in Table 1.

**Table 1:** Characteristics of included studies in the meta-analysis.

**References**	**Region**	**Diabetes types**	**Detecting methods**	**Specimen types**	**Case group (N)/control group (N)**	**Male (%) (DR/DM/healthy)**	**Age (years) (range/mean ±SD) (DR/DM/healthy)**	**BMI (kg/m^**2**^) (mean ±SD) (DR/DM/healthy)**	**NOS**
Yanko et al. (23)	Israel	Both	NA	Serum	DR (64)/DM (104)	100/100/	>40	NA	5
Olukoga et al. (28)	Nigeria	Both	Colorimetric	Plasma	DR (30)/DM (145)+Healthy (114)	//48.2	20–70	//24.52 ± 4.82	6
Weitzman et al. (29)	Israel	2	NA	Serum	DR (124)/DM (367)	NA	65 ± 9.4/64.1 ± 0.7/	28.4 ± 3.9/28.8 ± 4.8/	7
Huang et al. (30)	Taiwan	2	Colorimetric	Serum	DR (91)/DM (166)+Healthy (204)	//43.1	//58.2 ± 12.2	NA	5
Cai et al. (31)	China	2	Colorimetric	Serum	NPDR (59)+PDR (28)/DM (103)	48.3/49.5/	61.7 ± 17.4/53.6 ± 13.6/	24.6 ± 3.7/25.1 ± 3.5/	6
Navin et al. (32)	India	Both	Colorimetric	Serum	NPDR (21)+PDR (13)/DM (30)+Healthy (30)	NA	NA	NA	4
Longo-Mbenza et al. (33)	DR Congo	2	NA	Serum	DR (66)/DM (84)+Healthy (45)	39.4/46.4/46.7	53.4 ± 13.6/56.6 ± 12.4/50.7 ± 13.0	25.2 ± 5/26.3 ± 5.0/22.4 ± 2.9	6
Xia et al. (34)	China	2	HPLC	Plasma	NPDR (39)/DM (35)+Healthy (41)	53.8/57.1/55	56.5 ± 5.4/55.87 ± 7.0/54.4 ± 5.4	25.2 ± 4.0/25.1 ± 2.6/	7
Chuengsamarn et al. (35)	Thailand	2	Colorimetric	Serum	DR (154)/DM (452)	NA	NA	NA	7
Venkatachalam et al. (36)	India	2	Colorimetric	Serum	NPDR (10)+PDR (15)/DM (25)+Healthy (50)	64/56/46	64.6 ± 8.8/57.6 ± 7.8/52.9 ± 6.9	NA	5
Cui et al. (37)	China	2	Colorimetric	Serum	DR (141)/DM (1,608)	55.5/55.6/	57.1 ± 10.3/55.9 ± 11.3/	25.9 ± 2.9/26.3 ± 3.7/	8
Zhang et al. (38)	China	2	Colorimetric	Serum	DR (533)/DM (209)	56.3/56.9/	59.7 ± 10.5/59.2 ± 10.9/	25.1 ± 2.6/24.9 ± 3.4/	7
Pilemann-Lyberg et al. (39)	Denmark	1	Colorimetric	Plasma	NPDR (277)+PDR (229)/DM (142)	NA	NA	NA	7
Melo et al. (20)	Brazil	1	Colorimetric	Serum	DR (589)/DM (1,055)	58.2/54.4/	35.8 ± 11.6/26.9 ± 11.1/	25.1 ± 4.7/23.7 ± 3.8/	8
Chen et al. (40)	China	2	Colorimetric	Serum	NPDR (184)+PDR (162)/DM (172)	52.0/51.2/	52.8 ± 11.8/49.2 ± 8.5/	23.2 ± 3.5/23.1 ± 1.6/	7
Xia et al. (22)	China	2	Colorimetric	Serum	NPDR (582)+PDR (135)/DM (2,244)	46.4/52.0/	62.0 ± 10.0/60.0 ± 11.0/	NA	8
Shawki et al. (41)	Egypt	Both	Colorimetric	Serum	DR (70)/DM (40)+Healthy (40)	43/35/30	43.0 ± 10.7/45.4 ± 15.1/42.6 ± 9.4	32.4 ± 6.9/31.7 ± 6.7/30.8 ± 5.9	6
Çakir et al. (42)	Turkey	Both	Colorimetric	Serum	DR (68)/DM (54)+Healthy (42)	NA	63.4 ± 11.8/61.5 ± 11.2/59.3 ± 10.1	NA	5
Wakasugi et al. (43)	Japan	2	NA	Serum	NPDR (183)+PDR (39)/DM (777)	56.8/62/	66.0 ± 9.0/64.1 ± 9.8/	24.7 ± 3.9/24.6 ± 3.8/	7
Nakayama et al. (44)	Japan	2	Colorimetric	Serum	NPDR (72)/DM (142)	43/62/	64.0 ± 13.0/63.0 ± 10.0/	25.6 ± 4.5/25.6 ± 4.2/	7
Zhao et al. (45)	China	2	NA	Serum	NPDR (239)+PDR (104)/DM (75)	65/52/	51.7 ± 15.4/51.8 ± 14.3/	NA	6

*DM, Diabetes mellitus (without diabetic retinopathy when being used as controls); DR, diabetic retinopathy; PDR, proliferative diabetic retinopathy; NPDR, non–proliferative diabetic retinopathy; NDR, non–diabetic retinopathy; HPLC, high-performance liquid chromatography; BMI, body mass index; SD, standard deviation; DR Congo, The Democratic Republic of the Congo; NA, not available; NOS, Newcastle–Ottawa scale*.

### Comparison of UA Levels Between Patients With DR and Controls

**Figure 2:**
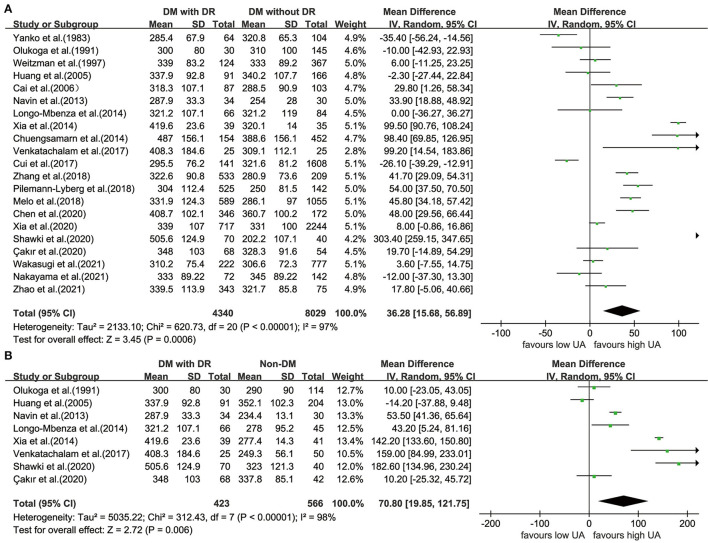
Forest plot for WMD and the corresponding 95% confidence interval of the comparison in UA levels between case group and control group with **(A)**/without **(B)** diabetes. WMD, weighted mean difference; UA, uric acid.

An obvious heterogeneity was observed among the 21 included studies (*I*^2^ = 97%; *P* < 0.001); thus, the random-effects model was used. We found that patients with DR had significantly higher UA levels UA than those in the controls with diabetes (WMD = 36.28; 95% CI: 15.68, 56.89; *P* < 0.001) (Figure 2A). Compared with healthy participants, the UA levels in patients with DR were higher (WMD = 70.80; 95% CI: 19.85, 121.75; *P* = 0.006; *I*^2^ = 98%; *P* < 0.001) (Figure 2B).

### Comparison of UA Levels Between Different Phases of DR and Controls With Diabetes

**Figure 3:**
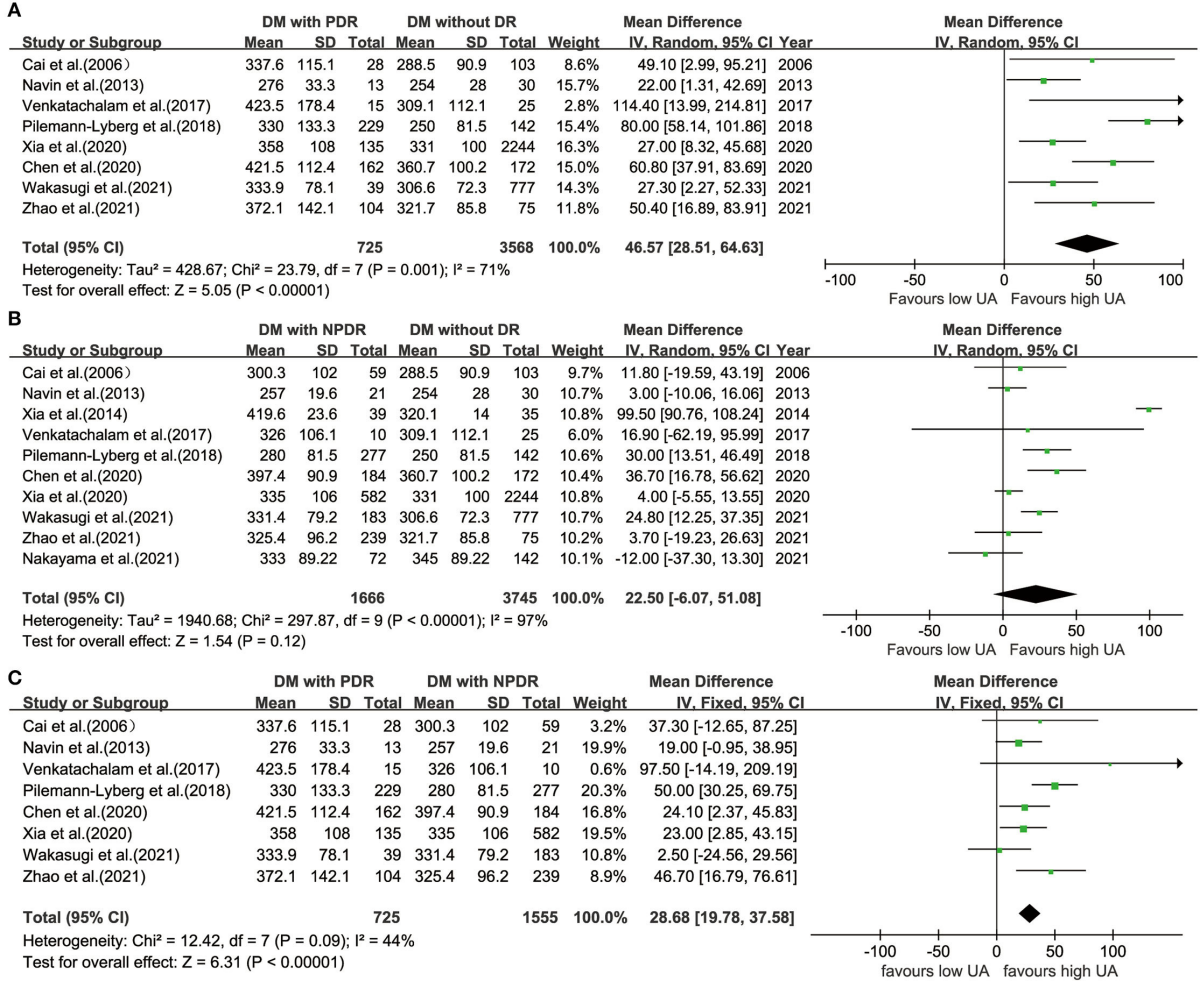
Forest plot for WMD and the corresponding 95% confidence interval of the comparison in UA levels between patients with PDR **(A)**/NPDR **(B)** and controls with diabetes, and between patients with NPDR and patients with PDR **(C)**. WMD, weighted mean difference; UA, uric acid; PDR, proliferative diabetic retinopathy; NPDR, non-proliferative diabetic retinopathy.

The UA levels were significantly higher in participants with PDR (WMD = 46.57; 95% CI: 28.51, 64.63; *P* < 0.001; *I*^2^ = 71%; *P* = 0.001) than those in the controls with diabetes (Figure 3A); however, when comparing UA levels in patients with NPDR and controls with diabetes, the difference is not statistically significant (WMD = 22.50; 95% CI:−6.07, 51.08; *P* = 0.120; *I*^2^ = 97%; *P* < 0.001) (Figure 3B).

### Comparison of UA Levels Between PDR and NPDR

Eight studies (22, 31, 32, 36, 39, 40, 43, 45) divided patients with DR into PDR and NPDR groups. There were significant differences in UA levels between patients with PDR and NPDR in a fixed-effects model (WMD = 28.68; 95% CI: 19.78, 37.58; *P* < 0.001; *I*^2^ = 44%; *P* = 0.090) (Figure 3C).

### Subgroup and Meta-Regression Analyses

**Figure 4:**
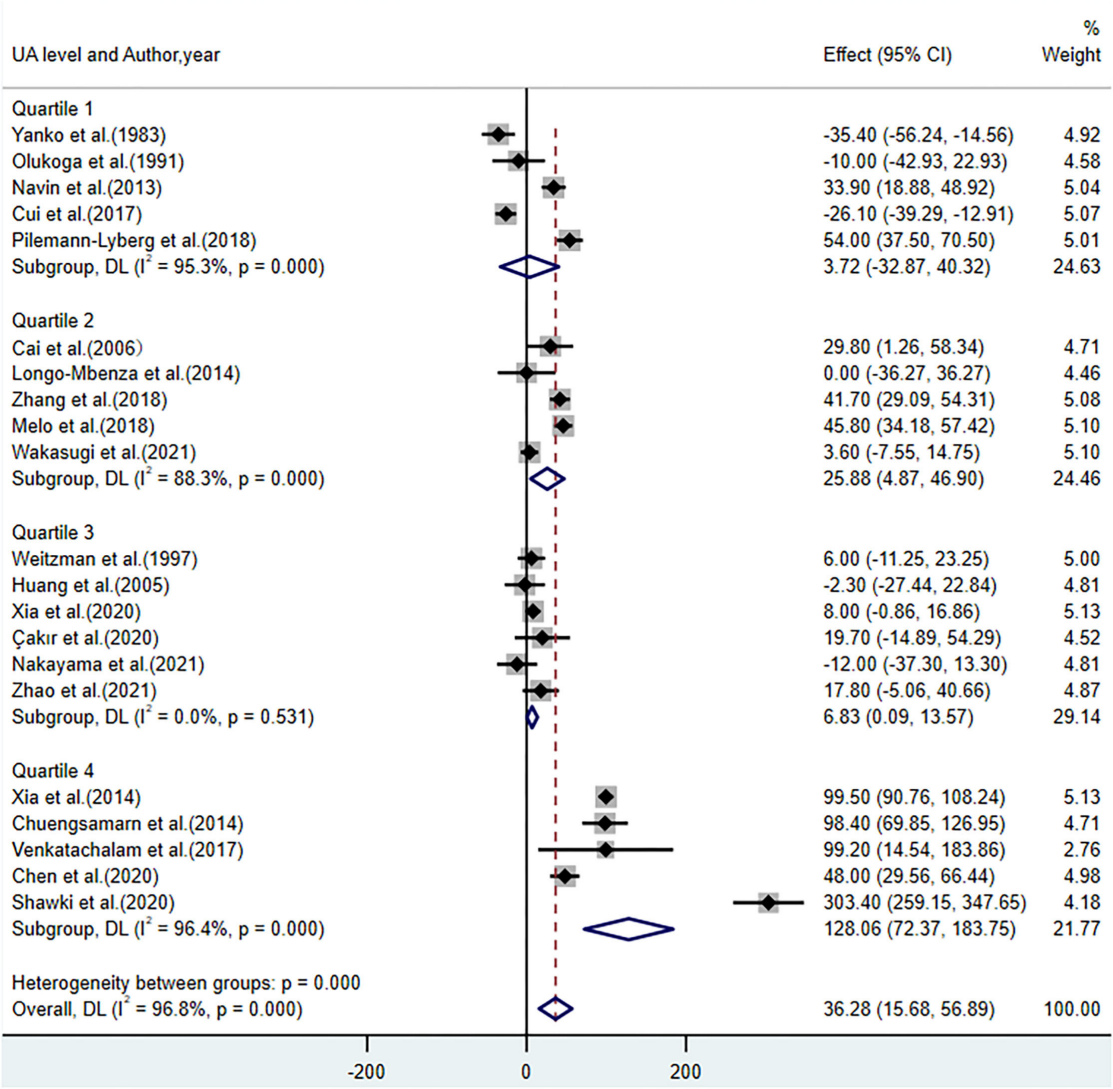
Forest plot of the subgroup analysis on the concentrations of UA in patients with DR compared with controls with diabetes. UA, uric acid; DR, diabetic retinopathy; Quartile 1 (285.4–307.1); Quartile 2 (307.1–333); Quartile 3 (333–378.15); Quartile 4 (378.15–505.6), μmol/L.

Table 2 presents the results of the subgroup analyses of UA levels between patients with DR and controls with diabetes. Most of the subgroup analysis results were consistent with the overall meta-analysis results, suggesting that these results were relatively stable but with high heterogeneity. Subgroup analyses of the region and diabetes type reported that UA levels were lower in Asians (WMD = 24.67; 95% CI: 2.30, 47.03; *P* = 0.031) and participants with type 2 diabetes (WMD = 27.16; 95% CI: 2.61, 51.71; *P* = 0.030). Increased UA levels were not significant in studies including both types of diabetes (WMD = 60.51; 95% CI: −19.27, 140.29; *P* = 0.137) and studies using plasma for UA measurement (WMD = 50.15; 95% CI: −1.42, 101.72; *P* = 0.057). When stratified by quality score (NOS <7 and NOS ≥7), the results showed that the heterogeneity failed to decrease in studies where NOS <7 with an *I*^2^ of 95.5%, and in NOS ≥7 studies, the *I*^2^ was 97.6%. The results were statistically significant in NOS <7 (WMD = 42.26; 95% CI: 1.14, 83.38; *P* = 0.044) and NOS ≥7 (WMD = 33.15; 95% CI: 7.45, 58.84; *P* = 0.011). Further subgroup analyses demonstrated increased UA levels in participants with a longer duration of diabetes (WMD = 62.22; 95% CI: 19.16, 105.27; *P* = 0.005), higher BMI (WMD = 63.51; 95% CI: 13.11, 113.91; *P* < 0.001), FBG (WMD = 52.76; 95% CI: 10.15, 95.37; *P* = 0.015), HbA_1c_ (WMD = 55.35; 95% CI: 23.92, 86.78; *P* = 0.001), and LDL (WMD = 55.39; 95% CI: 37.11, 79.67; *P* < 0.001). In addition, we divided the UA concentrations in patients with DR [median: 333; interquartile range (IQR): 307.1–378.15, mg/dL] by quartile. The subgroup analysis of UA levels showed an insignificant difference in quartile 1 (WMD = 3.72; 95% CI: −32.87, 40.32; *P* = 0.842), while in quartiles 2–4, especially in quartile 4, there was an increase in UA levels (WMD = 128.06; 95% CI: 72.37, 183.75; *P* < 0.001) (Figure 4).

**Table 2:** Subgroup analysis of the studies for the UA levels and DR.

				**Heterogeneity**	
**Subgroups**	**Number of studies**	**WMD (95% CI)**	***P***	***I* ^2^**	***P***
**Region**
Asia	16	24.67 (2.30; 47.03)	0.031	96.7%	<0.001
Others	5	76.30 (15.03; 137.57)	0.015	97.3%	<0.001
**DR phase**
NPDR vs. DM without DR	10	22.50 (−6.07; 51.08)	0.120	97.0%	<0.001
PDR vs. DM without DR	8	46.57 (28.51; 64.63)	<0.001	70.6%	0.001
PDR vs. NPDR	8	28.68 (19.78; 37.58)	<0.001	43.6%	0.088
**Diabetes types**
1	2	48.52 (39.02; 58.02)	<0.001	0.0%	0.426
2	14	27.16 (2.61; 51.71)	0.030	96.9%	<0.001
Both	5	60.51 (−19.27; 140.29)	0.137	97.9%	<0.001
**Duration of diabetes, years**
≤ 15	10	36.52 (4.81; 68.23)	0.024	97.6%	<0.001
>15	6	62.22 (19.16; 105.27)	0.005	97.7%	<0.001
**BMI, kg/m** ^**2**^
≤ 25	7	24.92 (8.14; 41.69)	0.004	89.5%	<0.001
>25	8	63.51 (13.11; 113.91)	0.014	98.4%	<0.001
**FBG, mg/dL**
≤ 150	5	47.47 (−0.62; 95.56)	0.053	98.1%	<0.001
>150	7	52.76 (10.15; 95.37)	0.015	97.4%	<0.001
**HbA**_**1c**_ **, %**
≤ 8.0	5	26.07 (2.62; 49.52)	0.029	90.4%	<0.001
>8.0	11	55.35 (23.92; 86.78)	0.001	97.4%	<0.001
**LDL, mg/dL**
≤ 120	12	42.93 (13.78; 72.09)	0.004	97.9%	<0.001
>120	5	53.39 (37.11; 79.67)	<0.001	79.2%	0.001
**Specimen types**
Plasma	3	50.15 (−1.42, 101.72)	0.057	96.4%	<0.001
Serum	18	32.77 (13.99; 51.55)	0.001	94.9%	<0.001
**UA level**, **μmol/L**
Quartile 1 (285.4–307.1)	5	3.72 (−32.87; 40.32)	0.842	95.3%	<0.001
Quartile 2 (307.1–333)	5	25.88 (4.87; 46.90)	0.016	88.3%	<0.001
Quartile 3 (333–378.15)	6	6.83 (0.09; 13.57)	0.047	0.0%	0.531
Quartile 4 (378.15–505.6)	5	128.06 (72.37; 183.75)	0.001	96.8%	<0.001
**NOS**
<7	10	42.26 (1.14; 83.38)	0.044	95.5%	<0.001
≥7	11	33.15 (7.45; 58.84)	0.011	97.6%	<0.001

*DR, diabetic retinopathy; PDR, proliferative diabetic retinopathy; NPDR, non–proliferative diabetic retinopathy; NDR, non–diabetic retinopathy; BMI, body mass index; SD, standard deviation; NOS, Newcastle–Ottawa scale; HbA1c, glycated hemoglobin A1c; UA, Uric acid 1 mg/dL = 59.48 μmol/L; FBG, fasting blood glucose 1 mmol/L =18.0 mg/dL; LDL, low-density lipoprotein 1 mmol/L= 38.66 mg/dL*.

According to the findings of subgroup analyses, a univariate meta-regression analysis regarding the clinical characteristics of participants, including BMI, duration of DM, FBG, HbA_1c_, and LDL, was performed to identify possible impact factors on the relationship between UA and DR. The results showed that BMI (*P* = 0.007, Adj *R*^2^ = 40.12%) and FBG levels (*P* = 0.040, Adj *R*^2^ = 29.72%) could explain the variation in study results, whereas the duration of DM (*P* = 0.099, Adj *R*^2^ = 14.93%), LDL (*P* = 0.308, Adj *R*^2^ = 0.61%), and HbA_1c_ (*P* = 0.537, Adj *R*^2^ = −5.43%) were not significant for determining the source of heterogeneity.

### Sensitivity Analysis and Publication Bias

**Figure 5:**
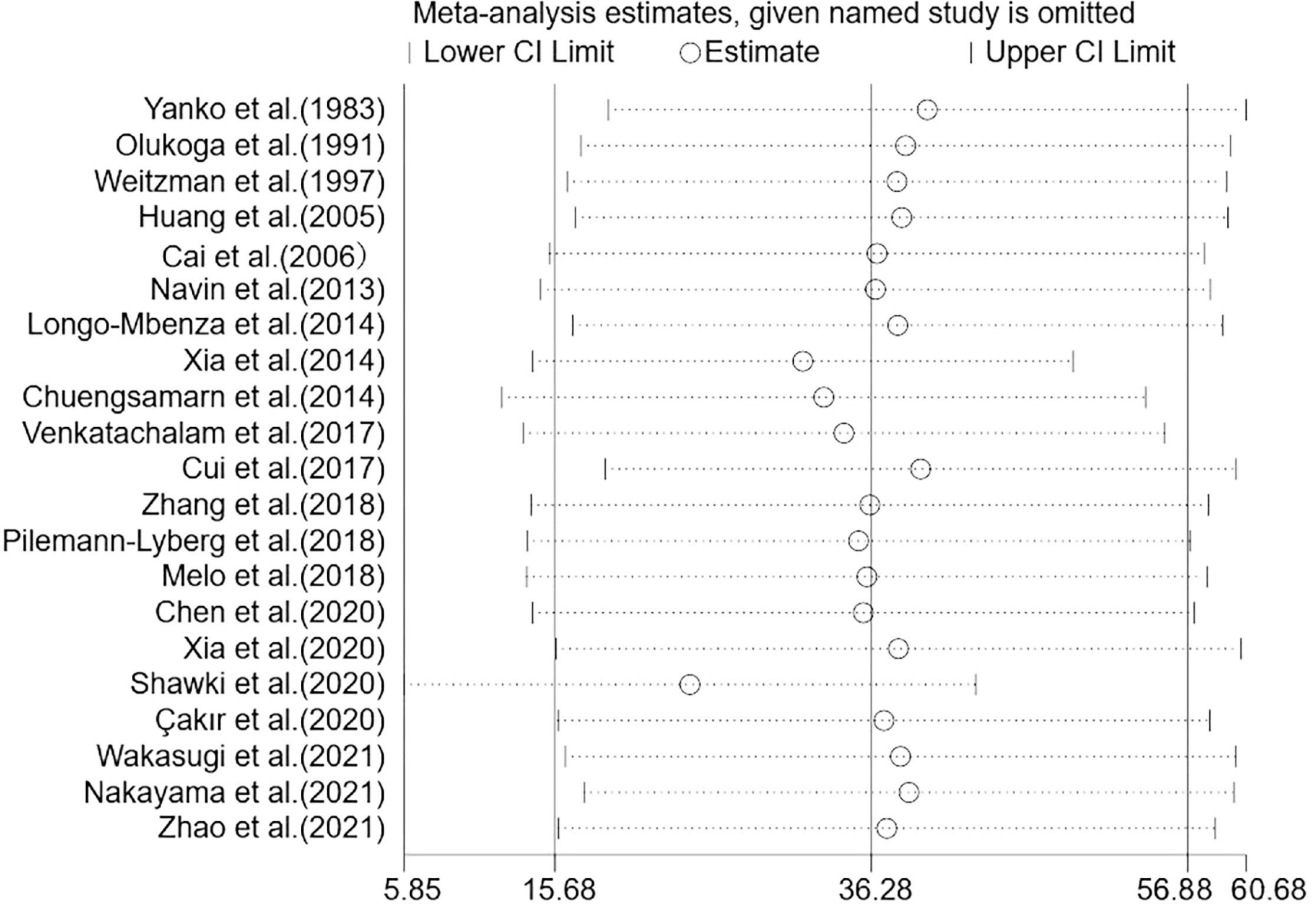
Sensitivity analysis of the 21 studies. Sensitivity analysis was performed according to omit one study in each turn. CI, confidence interval.

To evaluate the stability and reliability of our results, we performed a sensitivity analysis that excluded one study from the meta-analysis. After the included studies were successively removed, the estimates were statistically significant with WMD ranging from 24.46 (95% CI: 5.85, 43.07) to 39.91 (95% CI: 19.15, 60.68), indicating that the overall results were relatively stable (Figure 5). Notably, there was a marked decrease (though still obvious) in heterogeneity among studies when two sensitive studies (34, 41) were removed (WMD = 19.50; 95% CI: 5.87, 33.12; *P* = 0.005; *I*^2^ = 91%; *P* < 0.001), suggesting these two studies contributed relatively more to the heterogeneity.

The Egger funnel plot of the results of the included studies was symmetrical (Supplementary Material). The *P*-values of Begg's and Egger's tests of publication bias analyses were 0.291 and 0.156, respectively, suggesting that statistically significant publication bias.

## Discussion

To provide a better understanding of the relationship between UA and DR. We conducted a systematic review and meta-analysis to compare differences in UA levels between patients with DR and controls. We also tested whether UA levels could differ in different phases of DR, including NPDR and PDR. To the best of our knowledge, this is the first meta-analysis to show an exact association using MD with 95% CIs.

The results of our meta-analysis showed that UA levels in patients with DR were significantly higher than those in the controls. For different phases of DR, UA levels increased significantly in participants with PDR than those in the controls with diabetes. No significant difference was found in patients with NPDR (WMD = 22.50; 95% CI: −6.07, 51.08; *P* = 0.120; *I*^2^ = 97%; *P* < 0.001), while the existing heterogeneity possibly influenced the robustness of this result. In the comparison between patients with NPDR and controls, we noted that the study conducted by Xia et al. (34) used different specimens and detection methods for UA measurement. When this sensitive study was removed, the heterogeneity among the studies decreased sharply, and the difference was statistically significant (WMD = 13.50; 95% CI: 3.12, 23.89; *P* = 0.010; *I*^2^ = 65%; *P* = 0.003). In addition, increased UA levels existed in patients with PDR compared with patients with NPDR in our study, with no significant heterogeneity. This finding is consistent with previous studies showing that participants with higher UA levels have an increased risk of DR severity (from NPDR to PDR) (40, 51). Furthermore, a linear dose-response correlation of the elevation in patients with DR with different UA levels revealed a gradual increase from insignificant to significant. This is in line with the epidemiological survey showing that higher UA levels (≥378.00 μmol/L) were associated with a greater risk for DR (OR: 3.42; 95% CI: 1.64, 7.14; *P* = 0.001) (52), suggesting that elevated UA may be a potential risk factor for the progression of DR.

Increased UA is likely to play a role in the pathogenesis of DR. Accumulating experimental and clinical studies have found that oxidative stress and inflammatory responses induced by UA contribute to microvascular damage in DR (53, 54). Circulating UA is regarded as a powerful antioxidant that can remove superoxide and hydroxyl radicals in plasma, which may lead to an increase in reactive oxygen species production, which has been proven to cause coagulation disorders in the microcirculation (55). Furthermore, previous studies have demonstrated that UA could activate the NLRP3/NALP3 inflammasome and increase the expression of inflammatory factors such as TNF-α, IL-6, and CRP (17, 56). Several meta-analyses have shown higher levels of these inflammatory factors in patients with DR than those without DR (18, 57, 58). These inflammatory mediators have been shown to induce vessel dilation, retinal edema, platelet aggregation, and other pathological changes at the onset of DR (59, 60). Moreover, UA-lowering therapy has been confirmed to significantly decrease retinal and plasma levels of inflammatory cytokines and adhesion factors in streptozotocin-induced diabetes in rats (61). The role of anti-vascular endothelial growth factor (anti-VEGF) agents in targeting inflammation treatment to slow down the progression of DR has recently been regarded as effective (62). In addition, patients with diabetes with decreased urine UA excretion have been reported to have an increased risk of DR (63).

Since obvious heterogeneity existed among the 21 studies, it was imperative to explore the sources of heterogeneity. In subgroup analyses, high heterogeneity still existed. When stratified by quality score, the results showed that the heterogeneity failed to decrease, and the pooled results for each subgroup were approached. Notably, in subgroup analyses based on participants' clinical characteristics, such as duration of diabetes, FBG, and HbA_1c_, increased UA levels were observed in patients with DR with relatively poor health status. Further meta-regression analyses revealed that heterogeneity existed in the participants included in each study with different BMI and FBG levels. Considering the critical role of BMI and FBG levels in diabetes management (64, 65), it is rational to regard the severity of diabetes as the underlying source of heterogeneity. Possibly due to the differential therapies and health care services received by participants, diabetes severity was unevenly distributed across the included studies. For instance, unlike in developed regions, studies conducted in less-developed regions show higher UA levels (29, 41). Moreover, two sensitive studies (34, 41) were defined contributing more to the heterogeneity in sensitivity analysis.

Present meta-analysis had several limitations that may have affected the final conclusions. First, we failed to infer the causality of this association because of uncertainty about the temporal order. Evidence suggests that DR increases the risk of hyperuricemia in patients with diabetes (66). By summarizing the results of existing studies, we found that sex-related differences in this association remain unclear and deserve to be further elucidated. A previous cohort study reported an increased risk of newly developed DR in women [hazard ratio (HR): 2.17; 95% CI: 1.40, 3.37; *P* < 0.001] but not in men (HR: 1.08; 95% CI: 0.71, 1.66; *P* = 0.998) (19). However, Yanan Hu et al. investigated the association between UA and VTDR, showing that no sex-related difference was observed in the effect of UA on an increased risk of VTDR after adjustment (21). In addition, only a few studies have been conducted on patients with type 1 DM (20, 39, 67), which restricts the interpretation of results. Second, since the individual's continuous data, such as concentrations of UA, FBG, and BMI levels, were unavailable in each study, there were certain deviations for the subgroup analysis by transforming continuous variables into binary variables using the mean. Finally, the possibility of selection and unidentified confounding biases cannot be excluded. For example, the use of anti-hyperuricemic medications could be a potential confounder. A previous study showed that anti-hyperuricemic drugs are protective against retinal inflammation (61). However, most of the studies included in the meta-analysis did not control the use of anti-hyperuricemic medication; therefore, they possibly enrolled participants receiving UA-lowering therapy, which would limit the rigor of our results. In addition, similar to UA, homocysteine (Hcy) plays an important role in evoking oxidative stress (68), and Hcy levels are physiologically closely related to UA (69). Previous studies have also provided evidence that Hcy may also lead to endothelial injury in the retinal microvasculature at higher levels (70); this confounding factor needs to be recognized equally. Within these limitations, more prospective studies of high quality deserve launching to further confirm the association.

## Conclusions

In conclusion, our study provides evidence that UA levels are higher in patients with DR than those in the controls, but this difference is not statistically significant in the early phases. UA might be a potential biomarker for identifying disease severity in patients with DR rather than predicting the onset of DR among patients with diabetes. However, more prospective and high-quality clinical evidence is required to confirm these findings.

## Data Availability Statement

The original contributions presented in the study are included in the article/Supplementary Material, further inquiries can be directed to the corresponding author/s.

## Author Contributions

YCG and HLX contributed to the conception and design of this study. YCG and SYL performed the critical appraisal and data extraction. YCG was responsible for the subsequent analysis, interpretation of the data used in the systematic review, and meta-analysis. YCG drafted the manuscript. HLX and YCG revised it critically for important intellectual content. All authors have checked and approved this version to be submitted and finally published.

## Conflict of Interest

The authors declare that the research was conducted in the absence of any commercial or financial relationships that could be construed as a potential conflict of interest.

## Publisher's Note

All claims expressed in this article are solely those of the authors and do not necessarily represent those of their affiliated organizations, or those of the publisher, the editors and the reviewers. Any product that may be evaluated in this article, or claim that may be made by its manufacturer, is not guaranteed or endorsed by the publisher.
